# Synergistic hybridization of zinc sulfide, N-rGO, and polyaniline for enhanced energy and power density in asymmetric supercapacitors

**DOI:** 10.1039/d5ra08969g

**Published:** 2026-03-02

**Authors:** Maria Sadiq, M. U. Islam, Javed Ahmad, Maryam Hina, Mamoona Nawaz, Muhammad Salman

**Affiliations:** a Institute of Physics, Bahauddin Zakariya University Multan Pakistan dr.misbahulislam@bzu.edu.pk javedahmad@bzu.edu.pk; b Department of Physics, Quaid-i-Azam University Islamabad Pakistan

## Abstract

The depletion of non-renewable energy resources has intensified the need for advanced energy storage materials, with metal sulfides emerging as particularly promising candidates for supercapacitor applications. This study presents a systematic investigation of zinc sulfide (ZnS)-based nanocomposites, including binary composites with nitrogen-doped reduced graphene oxide (ZnS/NrGO, denoted as ZnG) and polyaniline (ZnS/PANI, denoted as ZnP), as well as a ternary composite (ZnS/NrGO/PANI, denoted as ZnPG). These materials were synthesized *via* a combined hydrothermal and *in situ* polymerization approach to optimize their electrochemical properties for supercapacitor applications. Structural characterization confirmed the cubic phase purity of ZnS, while morphological and elemental analyses (SEM-EDX) verified successful composite formation. Spectroscopic techniques (FTIR, XPS) elucidated the chemical bonding and electronic interactions within the materials. The ternary composite demonstrated superior electrical conductivity (*I*–*V* measurements) and a mesoporous architecture with an enhanced surface area of 168 m^2^ g^−1^ (BET analysis). Remarkably, electrochemical evaluations revealed outstanding performance metrics: a specific capacitance (*C*_s_) of 2645.94 F g^−1^, energy density (*E*_d_) of 111.2 Wh kg^−1^, and exceptional cycling stability (95.3% retention after 10 000 cycles). In the asymmetric device configuration, the ZnPG electrode delivered 1901.51 F g^−1^ at 2 A g^−1^, with *E*_d_ and power densities (*P*_d_) of 95.05 Wh kg^−1^ and 2201.21 W kg^−1^ respectively, demonstrating practical viability by powering an LED for 58 seconds. These results establish ZnPG as a highly efficient electrode material, showcasing the significant potential of metal sulfide-based nanocomposites for next-generation energy storage systems.

## Abstract 2

Hydrothermally synthesized ZnS/NrGO/PANI (ZnPG) ternary nanocomposite exhibits ultrahigh capacitance (2645.94 F g^−1^), high energy density (111.2 Wh kg^−1^), and excellent cycling stability (95.3%), enabling high-performance asymmetric supercapacitors for next-generation energy storage.

## Graphical abstract

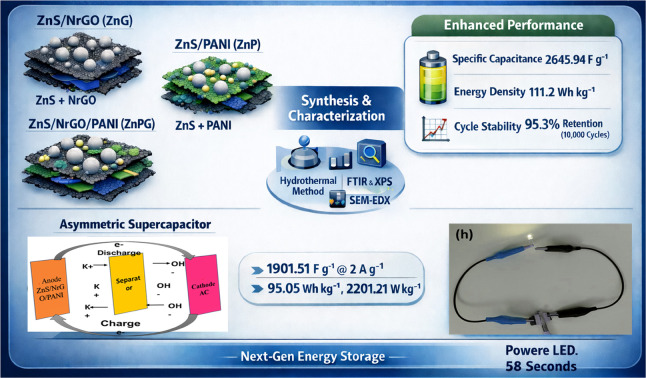

## 1. Introduction

Energy has become the critical foundation for economic growth and technological competitiveness in today's interconnected world.^1,2^ But there are pressing problems with the current energy situation that need fixing right now. These include the ever-increasing demand for energy, the exhaustion of our limited fossil fuel reserves, and the negative environmental effects of burning these fuels, like pollution and climate change.^3–5^ The transition to sustainable energy systems demands immediate interdisciplinary collaboration between materials scientists and engineers to develop next-generation energy storage technologies that are both economically viable and technologically robust.^6–8^ Energy storage plays a vital role in addressing the intermittency of renewable energy sources. Through dedicated research efforts, material researchers are working towards advancing energy storage technologies such as batteries and supercapacitors. These advancements are focused on enhancing energy storage capacity, extending cycle life, and improving cost efficiency. Supercapacitors are emerging as transformative solutions in energy storage due to their exceptional power density, rapid charging capabilities, and extended cycle life. These characteristics make them particularly well-suited for applications requiring short bursts of energy, such as in electric vehicles.^9^ By incorporating supercapacitors into energy systems, we can facilitate the integration of renewable energy, boost energy efficiency, and address the ongoing energy crisis, paving the way for a more sustainable and reliable energy future. Supercapacitors are classified into two types by their storage mechanism: electrochemical double-layer capacitors (EDLCs), which store charge electrostatically at the electrode–electrolyte interface, and pseudocapacitors, which store energy through surface redox reactions.^10,11^

Metal sulfides have emerged as particularly promising electrode materials among various candidates, including carbon-based materials (activated carbon, graphene), transition metal oxides (TiO_2_, RuO_2_, V_2_O_5_, MnO_2_), and conducting polymers (PPy, PANI).^12,13^ These materials offer an attractive combination of high specific capacitance, superior power density, and reversible faradaic redox activity. Their advantages are further enhanced by natural abundance, cost-effectiveness, and excellent structural stability, leading to exceptional cycling durability.^14,15^ Current research focuses on optimizing their electrochemical performance for advanced supercapacitor applications. NrGO has gained significant attention due to its excellent properties, including high surface area, superior electrical conductivity, remarkable chemical stability, and extended cycle life, making it an ideal candidate for high-performance energy storage systems.^16^ Polyaniline (PANI) has attracted considerable research interest as a supercapacitor material owing to its combination of high capacitance, facile processing characteristics, and reversible faradaic reactions – properties essential for advanced energy storage applications.^17,18^ Metal sulfide-based composites incorporating conducting polymers like Polyaniline (PANI) and NrGO have emerged as promising advanced electrode materials for supercapacitors to improve further the overall energy storage capacity, charge/discharge rates, and cycling stability.^19^ This synergistic combination of metal sulfides, conducting polymers, and carbonaceous materials in composite electrodes holds great potential for advancing the efficiency and practicality of supercapacitor technology.^20^ Extensive research efforts have been devoted to developing optimized PANI-based composite electrodes for supercapacitor applications. Athira *et al.* developed a PANI/Co_3_O_4_/rGO ternary composite *via in situ* chemical oxidative polymerization, achieving an exceptional specific capacitance of 1982 F g^−1^ at 10 mV s^−1^ while maintaining 80.2% capacitance retention after 5000 cycles.^21^ Further advancing this approach, Hamid Heydari *et al.* synthesized PANI/rGO/CoS electrodes through hydrothermal methods, obtaining a specific capacitance of 431 F g^−1^ at 0.5 A g^−1^ in acidic electrolyte with outstanding cyclic stability (90.1% retention after 1000 cycles).^20^ These studies highlight the potential of carefully engineered PANI-based ternary composites for high-performance supercapacitor electrodes.^20^

Despite the growing interest in advanced electrode materials, limited research has focused on exploring metal sulfides for energy storage applications. This gap in the literature motivated our investigation into ZnS-based nanocomposites for supercapacitor electrodes. In this work, we report for the first time the synthesis of ZnS and its binary/ternary composites with carbonaceous materials (PANI and NrGO) *via* a facile hydrothermal approach. These novel materials are anticipated to exhibit enhanced electrochemical properties, offering significant potential for advancing next-generation energy storage technologies. ZnPG composite ZnS nanoparticles on the NrGO sheets are uniformly covered in PANI matrix to create a conduction network and pseudocapacitance network, which are effective pathways of transporting electrons and ions. The large numbers of heterointerfaces allow charge storage of both EDLC and faradaic and structural integrity is maintained by the flexible matrix of PANI. This synergetic architecture leads to a higher performance of electrochemical performances than individual and binary components.

## 2. Materials and methods

### 2.1 Reagents

Sulfuric acid (H_2_SO_4_, 98%), potassium permanganate (KMnO_4_, ≥99%), graphite powder (≥99.5%), sodium nitrate (NaNO_3_, ≥99%), hydrogen peroxide (H_2_O_2_, 30 wt%), l-ascorbic acid (C_6_H_8_O_6_, ≥99%), ammonium fluoride (NH_4_F, ≥98%), sodium hydroxide (NaOH, ≥98%), zinc nitrate hexahydrate (Zn(NO_3_)_2_·6H_2_O, ≥99%), sodium sulfide hydrate (Na_2_S·*x*H_2_O, ≥98%), hydrochloric acid (HCl, 37%), ammonium persulfate ((NH_4_)_2_S_2_O_8_, ≥98%), and aniline (C_6_H_5_NH_2_, ≥99.5%) were purchased from Sigma-Aldrich and used as received without further purification. Ethanol (C_2_H_5_OH, ≥99.9%) was obtained from Merck. Deionized water (DIW) was used throughout all experiments.

### 2.2 Synthesis of ZnS, ZnG

**Fig. 1:**
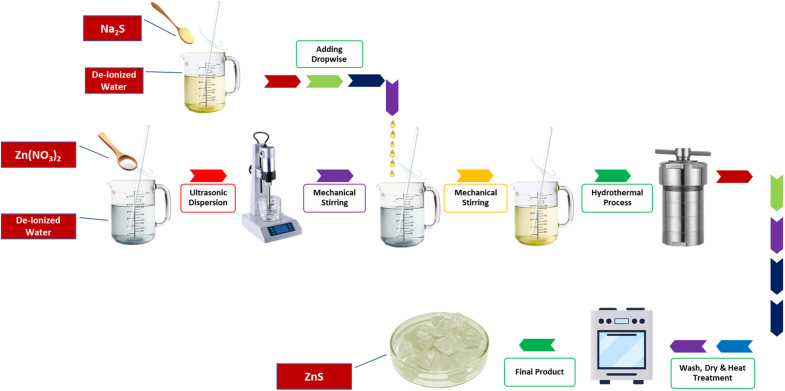
Schematic diagram of synthesis for ZnS.

The NrGO synthesis followed our previously reported method.^22^ For nanocomposite preparation, 15 wt% NrGO was dispersed in 50 ml DI water *via* one hour of sonication. Separately, 2 M Zn(NO_3_)_2_·6H_2_O solution (30 ml DI water) was prepared under 1 h of stirring. The NrGO dispersion was then combined with the zinc nitrate solution, followed by dropwise addition of 2 M Na_2_S under continuous stirring. After 1 h of homogenization, the mixture was hydrothermally treated at 140 °C for 8h in a Teflon-lined autoclave. The resulting ZnG precipitate was washed, vacuum-dried at 70 °C, and ground into powder. Pure ZnS was synthesized identically without NrGO addition. The optimized condition (2 M precursors, 140 °C for 12 h, and 15 wt% NrGO) was selected based on the formation of phase-pure ZnS without secondary impurities (XRD), uniform nanoparticle distribution on NrGO sheets (SEM), and superior electrochemical performance. The schematic diagram is illustrated in Fig. 1 and reaction is shown in eqn (1).

1 Na_2_S + Zn(NO_3_)_2_ → ZnS + 2NaNO_3_

### 2.3 Synthesis of ZnP, and ZnPG composites

The ZnP binary composite was synthesized *via in situ* polymerization, beginning with the dispersion of 1.3 g ZnS nanocomposite in 12 ml ethanol through 45 minutes of ultrasonication. The homogeneous suspension was then transferred to a round-bottom flask, where 1165 mg of ammonium persulfate (APS) oxidant was added, followed by the introduction of 688 µl aniline monomer under continuous stirring until complete dissolution. The reaction pH was carefully controlled through incremental addition of HCl solution. The polymerization process was conducted under optimized conditions, starting with 30 minutes of equilibration in an ice bath (0–4 °C) followed by 12 hours of continuous reaction at 70 °C with constant agitation. The resulting product was collected by vacuum filtration, thoroughly washed with DIW, and dried at 80 °C for 12 hours before being finely ground using an agate mortar and pestle. For the ternary ZnPG composite, a modified *in situ* polymerization route was employed to ensure uniform incorporation of NrGO within the PANI matrix. Initially, 1.3 g of the pre-synthesized ZnG nanocomposite was dispersed in 12 mL ethanol by ultrasonication for 45 minutes to achieve homogeneous distribution of ZnS nanoparticles on NrGO sheets. This suspension was transferred to a round-bottom flask, followed by the addition of 1165 mg APS oxidant. Subsequently, 688 µL of aniline monomer was added under continuous stirring, and the reaction pH was carefully adjusted using HCl solution. To encourage uniform PANI coating around the ZnG framework, the polymerization was started by keeping the reaction mixture in an ice bath at 0–4 °C for 30 minutes. This was followed by constant stirring at 70 °C for 12 hours. Before being ground into a fine powder, the resultant ZnPG composite was recovered by vacuum filtering, repeatedly cleaned with deionized water to get rid of unreacted species, and dried at 80 °C for 12 hours.

### 2.4 Electrode fabrication

The electrodes were fabricated *via* a drop-casting method, where a homogeneous slurry was prepared by thoroughly mixing active material, carbon black, and PVDF binder in *N*-methyl-2-pyrrolidone (NMP) solvent at a precise weight ratio of 95 : 5:5. The active material refers to the synthesized composite electrode material (ZnS, ZnG, ZnP, or ZnPG). This optimized slurry composition was then uniformly coated onto pre-cleaned nickel foam substrates (1 × 1 cm^2^) with careful control of the active material loading to 3 mg cm^−2^. The coated electrodes were subsequently dried at 80 °C for 6 hours in a vacuum oven to ensure complete solvent removal and proper adhesion of the active material to the current collector. 2 M KOH electrolyte was used for electrochemical analysis. A 2 M KOH aqueous electrolyte was prepared by dissolving an appropriate amount of analytical-grade potassium hydroxide pellets in DIW under continuous stirring until complete dissolution. The electrolyte solution was allowed to equilibrate to room temperature.

## 3. Characterization

The synthesized materials were comprehensively characterized using multiple analytical techniques to evaluate their physicochemical properties. Structural analysis was performed using X-ray diffraction (XRD) to confirm crystallinity and phase purity. Morphological features and elemental composition were examined through scanning electron microscopy (SEM) with energy dispersive X-ray spectroscopy (EDX). Electrical properties were assessed *via* current–voltage (*I*–*V*) measurements, while surface characteristics including specific surface area and porosity were determined by Brunauer–Emmett–Teller (BET) analysis. Thermal stability was investigated using thermogravimetric analysis (TGA).

## 4. Results and discussions

### 4.1 X-ray diffraction analysis

**Fig. 2:**
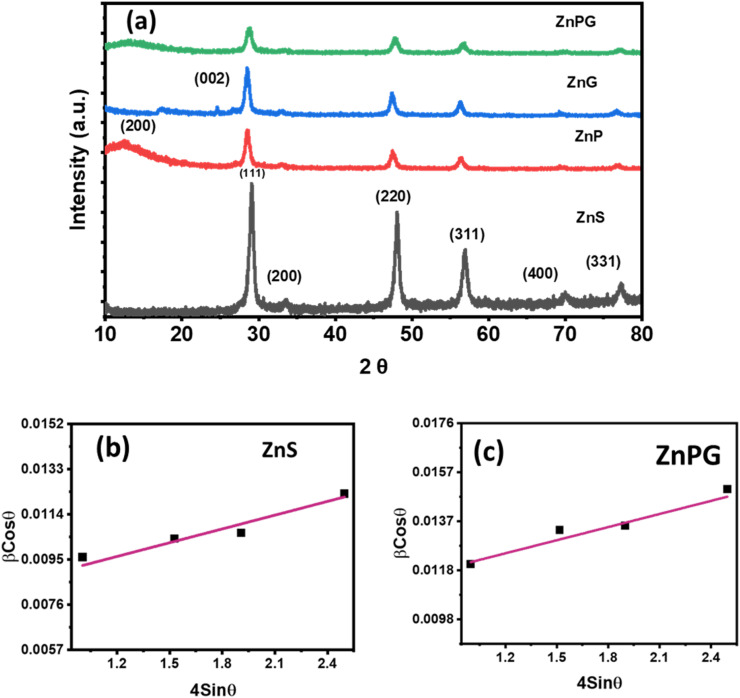
(a) XRD pattern (b and c) W–H plot of ZnS and ZnS/NrGO/PANI nanocomposites.

XRD analysis confirmed the crystalline structure of the synthesized materials as shown in Fig. 2. Pure ZnS displayed characteristic FCC-phase peaks (JCPDS 05-0566) at 2*θ* = 29.15° (111), 33.47° (200), 48.01° (220), 57.07° (311), 70.11° (400), and 77.31° (331), demonstrating high phase purity. In nanocomposites, the reduced peak intensity and slight shifts toward lower angles (∼0.3–0.5°) revealed successful incorporation of NrGO and PANI into the ZnS matrix, with concomitant grain size expansion. The absence of impurity peaks further validated phase purity,^26^ while peak broadening suggested PANI integration with the metal sulfide lattice.^22,27^ A significant drop in peak intensity is seen upon integration with NrGO and PANI, implying the effective inclusion of these components into the ZnS matrix and a likely decrease in crystallite size or increase in structural disorder. There was also a slight shift of the peaks towards lower diffraction angles, which might suggest lattice expansion resulting from the interaction with the carbon-based matrix and conducting polymer. The phase purity of the composites is confirmed even further by the absence of extra diffraction peaks linked to contaminants. The observed little peak widening and distortion are ascribed to PANI, which is known to affect the crystallinity and cause structural stress in the composite.

The other parameters, including lattice constants, volume, FWHM, and dislocation density, were estimated by Bragg's law.^28^ The slight variation in these parameters is due to ZnG interfacial interactions with PANI matrix. The observed lattice ion migration facilitates improved energy storage performance.^23^

Additionally, Williamson–Hall (W–H) plots were utilized to assess the strain levels, as depicted in Fig. 2(b and c). Based on these results, crystallite size shows a decrement trend for composites, confirming the compression inside the lattice structure.

Table 1 illustrates crystallite size, FWHM, and strain, utilizing Cerlef software. The following Scherer equation is employed to ascertain crystallite size.^24^2
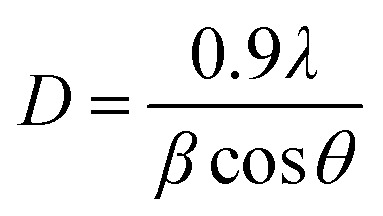
In this context, *D* represents the crystallite size (nm), *λ* stands for the wavelength (nm) *θ* is the angle of diffraction linked to the peak and *β* is the full width at half maximum (FWHM) in radians,. The grain size is calculated for every peak in the XRD pattern, and the average is obtained. As seen below, the dislocation density (*δ*) has an inverse relationship with *D*.^25^3
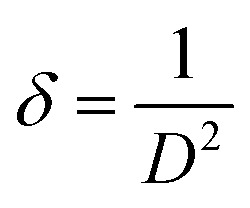


**Table 1:** Structural parameters for ZnS, ZnG, ZnP, and ZnP/NrGO nanocomposites

Materials	*a* (Å)	*b* (Å)	*c* (Å)	*V* (A^3^)	FWHM (*β*)	Crystallite size (nm)	Dislocation density	Strain *ε*_str_
ZnS	5.36	5.36	5.36	159.76	0.605	6.396	0.036	0.0099
ZnG	5.42	5.42	5.42	157.84	0.677	5.737	0.046	0.0103
ZnP	5.40	5.40	5.40	156.78	0.678	5.728	0.045	0.0108
ZnPG	5.39	5.39	5.39	153.75	0.773	5.219	0.057	0.0122

The observed lattice stresses remain constrained within a narrow range, showing only marginal variation across different material compositions. Dislocations form from coalescence processes, where smaller crystallites merge to form elongated clusters with redefined grain boundaries. Notably, the diminished crystallite size correlates with oxygen vacancy formation, while applied stress inhibits further nanoparticle growth. These structural defects promote charge carrier dispersion, significantly improving photocatalytic performance by suppressing electron–hole recombination. To validate the doping mechanism, sample strain was quantitatively analyzed using the Stokes–Wilson equation.

4 *ε*_str_ = (*β*/4)tan *θ*

Crystallite size and *ε*_str_ in the produced materials were studied using the Williamson–Hall (W–H) method. The W–H plot (Fig. 2b and c) for pure ZnS showed a well balanced contribution from crystallite size and lattice strain, with no peak broadening seen in the XRD pattern. On the other hand, when NrGO was added, significant *ε*_str_ rise and related crystallite size drop were seen. NrGO's flexible, conductive character causes lattice distortions and improves ion transport and charge transfer, hence improving the general electrochemical performance.^26^ It shows that the *ε*_str_ changes when NrGO and PANI are doped into ZnS. It lays the groundwork for the idea that the composite atoms are located in the host lattice's interstices.

### 4.2 Morphology analysis

**Fig. 3:**
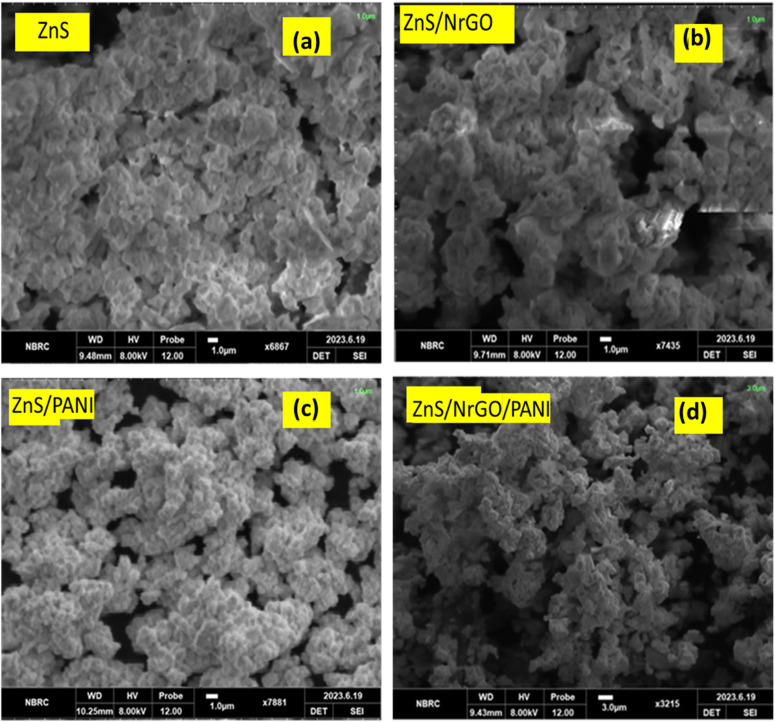
SEM images of (a) ZnS, (b) ZnG, (c) ZnP and (d) ZnPG nanocomposites.

The surface morphology of the synthesized composites, as examined by SEM at 1 µm magnification (Fig. 3), plays a crucial role in determining their electrochemical performance.^27^ Both binary (ZnG, ZnP) and ternary (ZnPG) composites exhibit porous architectures, with the ternary composite demonstrating particularly enhanced porosity due to the uniform growth of NrGO/PANI on the ZnS framework. This well-developed porous structure provides several advantages: it significantly increases the electroactive surface area for improved ion accessibility^5^ enhances mechanical stability through the synergistic combination of ZnG within the PANI matrix.^3^ and facilitates efficient charge transport due to the reduced nanoparticle size (30–50 nm) which creates additional active sites for redox reactions.^28^ The improved electrical conductivity can be attributed to the optimized interfacial contact and quantum confinement effects in the nanostructured composite.^29^

**Fig. 4:**
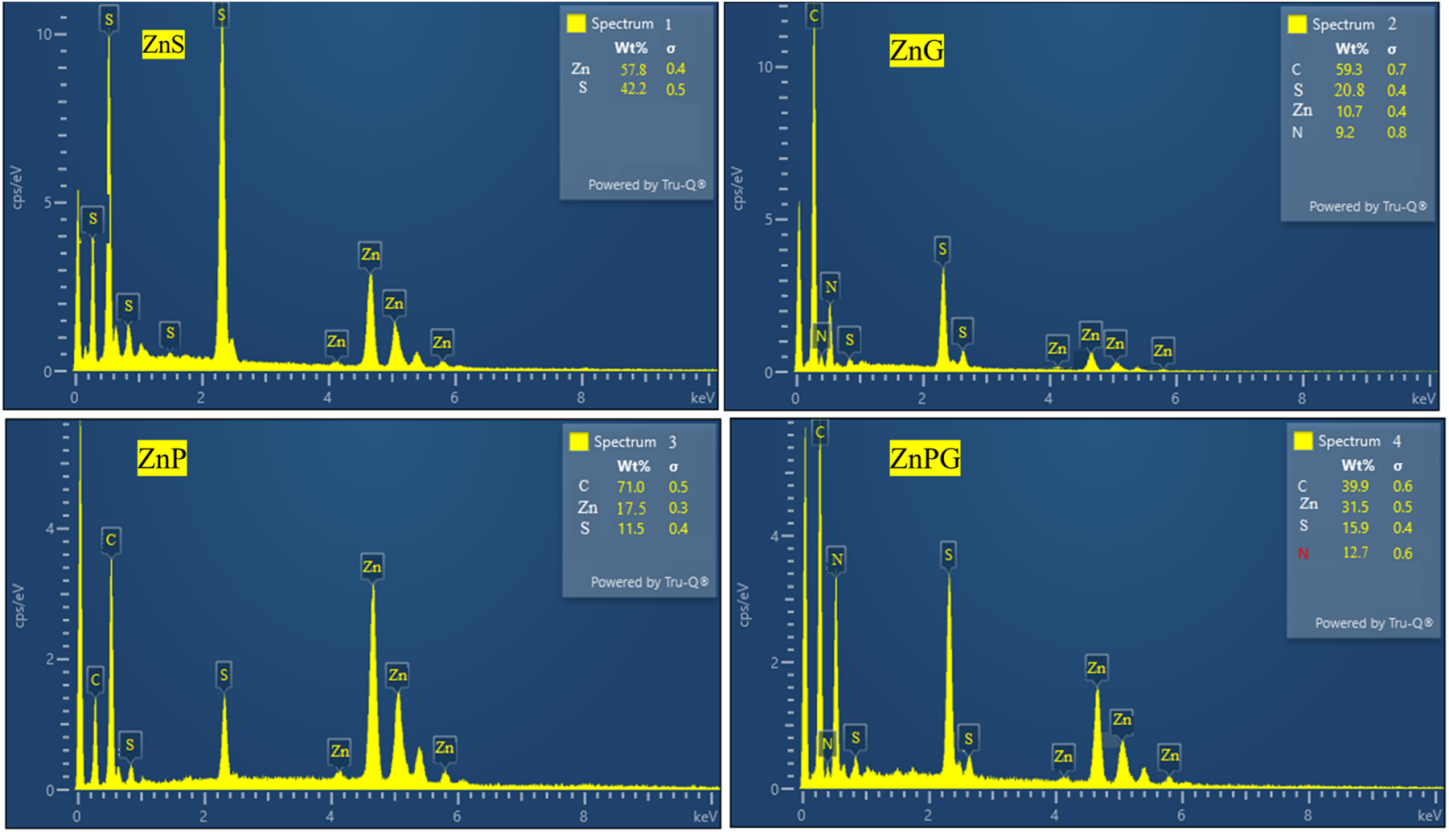
EDX pattern of ZnS, and all its nanocomposites.

EDX analysis (Fig. 4) confirmed the presence of Zn, N, C, and S in their expected stoichiometric ratios, with no detectable impurities, further validating the high phase purity of the materials as initially indicated by XRD results. This combination of morphological and compositional characteristics contributes to the superior electrochemical performance observed in these composites.

### 4.3 X-ray photoelectron spectroscopy (XPS)

**Fig. 5:**
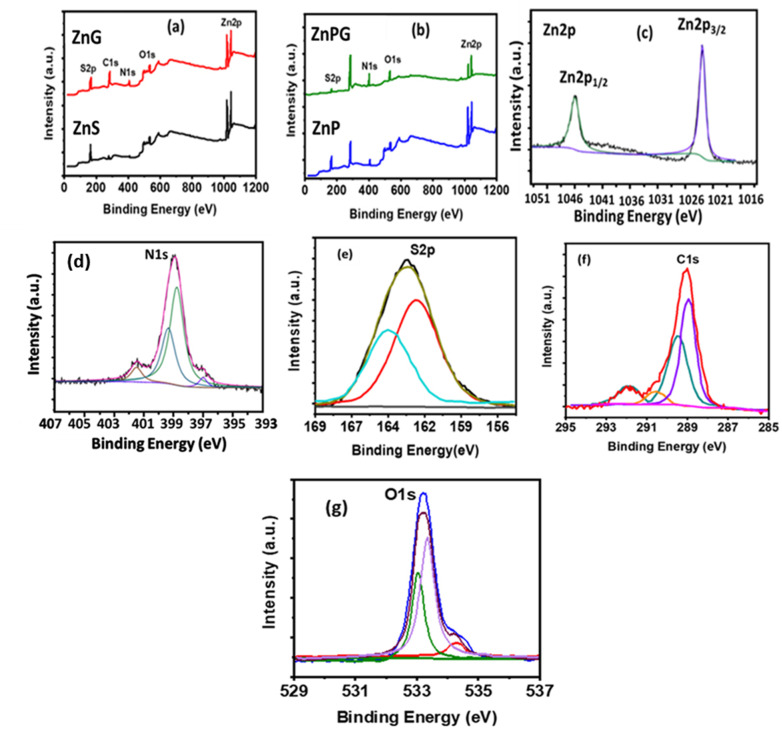
High-resolution XPS deconvolution spectra of (a) pristine ZnS and ZnG composite, (b) ZnP and ternary ZnPG composite, along with core-level spectra of (c) Zn 2p, (d) N 1s, (e) O 1s, (f) C 1s, and (g) S 2p.

The chemical composition and electronic states of the synthesized materials were thoroughly characterized by XPS (Fig. 5). The survey spectrum of pure ZnS exhibits characteristic peaks at 1023.1 eV (Zn 2p) and 160.93 eV (S 2p), confirming the presence of zinc and sulfur. For the ZnG composite , additional peaks emerge at 284.32 eV (C 1s), 399.96 eV (N 1s), and 532.43 eV (O 1s), along with shifted Zn 2p (1021.9 eV) and S 2p (161.51 eV) peaks, verifying successful NrGO incorporation.^30,31^ The ternary ZnPG composite (Fig. 5b) shows intensified C 1s and N 1s signals with attenuated Zn/S signals, demonstrating effective PANI integration that aligns with XRD results. High-resolution spectra reveal detailed chemical states, the Zn 2p region shows spin–orbit doublets (1022.2 eV for 2P_3/2_ and 1045.3 eV for 2P_1/2_) confirming Zn^2+^, The N 1 s spectrum confirms the incorporation of nitrogen functional groups such as pyridinic (399.4 eV), pyrrolic (400.5 eV), and graphitic nitrogen (400.1 eV).^32^ The high-resolution S 2p spectrum (Fig. 5e) displays two peaks at 162.3 eV (S 2p_3/2_) and 167.9 eV (S 2p_1/2_), indicating the presence of sulfur in two bonding states. The dominant peak at 162.3 eV suggests that S^2−^ is the primary sulfur species on the surface.Finally, the O 1 s spectrum (Fig. 5g) reveals four distinct peaks at 530.9 eV (O–Zn, indicating interaction between Zn^2+^ and oxygen atoms of NrGO), 531.9 eV (O=C), 532.9 eV (O–C=O), and 534.1 eV (O=S), further supporting the complex surface chemistry and strong interfacial interactions among the components. The C 1 s spectrum (Fig. 5f) is deconvoluted into four peaks at 289.3 eV (C=C, graphitic carbon), 290.9 eV (C–N), 292.9 eV (C–O), and 294.0 eV (C=O), confirming the diverse carbon bonding environments present in the composite structure.^33,34^

### 4.4 Vibrational spectroscopy

**Fig. 6:**
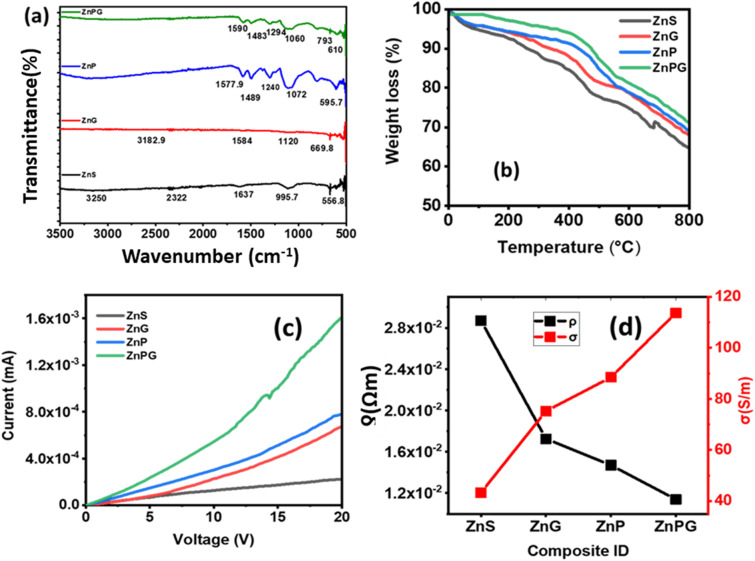
(a) FTIR spectra. (b) TGA plots (c) *I*–*V* graphs (d) resistivity and conductivity.

FTIR spectroscopy is a versatile analytical tool that enables the differentiation between diverse species and the identification of specific infrared bands associated with certain materials.^32^ The high-resolution spectra were recorded in the range of 500 to 4000 cm^−1^ to reveal the vibrational bands associated with different functional groups and molecular vibrations in the synthesized composites, as depicted in Fig. 6a. Notably, Zn–S stretching vibrations at 500 cm^−1^, S–S stretching at 556.8 cm^−1^, and S–S bending modes at 665.8 cm^−1^ were identified in the ZnS spectrum. The incorporation of NrGO led to the appearance of characteristic bands linked to C–N and C=C stretching modes, along with a broad band at 1120 cm^−1^, suggesting the strong formation of ZnS nanoparticles and successful bonding of certain Zn cations by NrGO.^35^ Further, the addition of PANI resulted to increased band intensity at 1240.02 cm^−1^ and 1399.2 cm^−1^, corresponding to C–N stretching and C=H bending modes, indicating the successful embedding of PANI into the ZnS matrix.

### 4.5 TGA analysis

TGA profile for pure ZnS and all nanocomposites are shown in Fig. 6(b). The thermograms of ZnS demonstrate the occurrence of two distinct stages of dehydration. The observed weight loss of around 7.3% within the temperature range of 25 °C to 800 °C can be linked to the loss of weakly bound and absorbed water. The following 8.9% weight loss up to 350 °C, on the other hand, is linked to the removal of the remaining firmly bound water involved in crystallisation in the dehydrated cubic phase.^36^ ZnS, ZnG, ZnP, and ZnPG show unique thermograms in terms of the degree of dehydration seen in the ZnS crystalline phase. Within the temperature range of 100 to 300 °C, which corresponds to the dehydration process of ZnS crystal, the observed weight loss is 6.41% for the ZnG and 4.8% for the ZnP and 2.9% for the ZnPG. This finding implies that as NrGO and PANI are added, the amount of crystal water in the ZnG and ZnP decreases. The ZnPG nano-hybrids show a continuous drop in weight beyond 450 °C, which might be attributable to graphene and PANI combustion within the nanocomposites.

### 4.6 Current–voltage (*I*–*V*) measurements

The voltage range from 0 V to 20 V was used to measure the *I*–*V* characteristics, as illustrated in Fig. 6c. A linear behavior was exhibited by all the synthesized samples, indicating their ohmic nature. However, ZnP/NrGO-based electrodes stand out with higher current values, indicating enhanced electrical conductivity and lesser resistivity, depicted in Fig. 6d. The reduced resistance is ascribed to the integration of PANI and NrGO inside the metal sulfides. The influence of contact resistance on energy storage devices renders the observed decrease in resistivity from the incorporation of NrGO in PANI matrix particularly advantageous for supercapacitor applications. Incorporating PANI and NrGO, which exhibit high conductivity, facilitates improved performance and efficiency.^34^

### 4.7 BET analysis

**Fig. 7:**
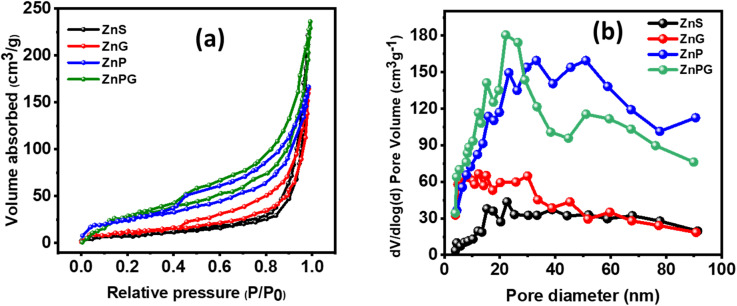
(a) N_2_ adsorption–desorption isotherms and (b) pore size distribution for all nanocomposites.

BET analysis was conducted to evaluate key structural parameters, including specific surface area, pore volume, and pore size distribution, which critically influence electrode performance. As shown in Fig. 7a, the N_2_ adsorption–desorption isotherms and corresponding data in Table 2 confirm the mesoporous nature of all samples. The ternary ZnPG composite exhibited the highest specific surface area among the tested materials (Fig. 7b), a characteristic that significantly enhances electrochemical performance through two primary mechanisms, shortened electrolyte diffusion pathways and increased electroactive sites for ion transport. This improved activity stems from the substantially expanding electrode–electrolyte interfacial contact area rather than bulk material properties, enabling more efficient charge storage and transfer processes.^37^

**Table 2:** Surface area, pore volume, and pore size for all synthesized samples

Materials	Surface area (m^2^ g^−1^)	Pore size (nm)	Pore volume (cm^3^ g^−1^)
ZnS	72	3.87	0.0041
ZnG	108	3.72	0.0039
ZnP	136	3.69	0.0031
ZnPG	168	3.62	0.0021

## 5. Electrochemical measurements

The electrochemical performance was systematically evaluated through cyclic voltammetry (CV) to assess redox behavior and capacitive properties, complemented by galvanostatic charge–discharge (GCD) measurements to determine specific capacitance, energy density, and power density. Charge transfer kinetics and interfacial properties were analyzed using electrochemical impedance spectroscopy (EIS), providing insights into electrolytic resistance and electrode–electrolyte interactions. An asymmetric supercapacitor device was assembled using ZnPG as the anode and activated carbon as the cathode, separated by a filter paper membrane. The current collectors consisted of copper foils electrochemically coated with gold to enhance conductivity. The device employed 2 M KOH aqueous electrolyte for optimal ionic transport.

### 5.1 CV measurements

**Fig. 8:**
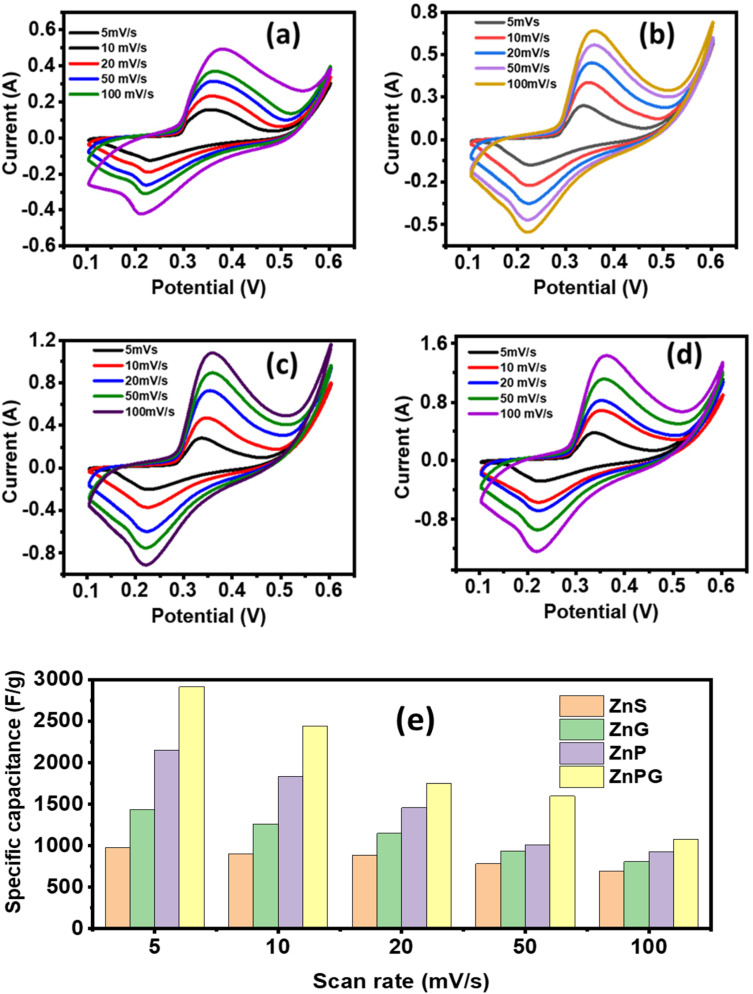
CV curve of (a) ZnS, (b) ZnG, (c) ZnP, (d) ZnPG composites. (e) *C*_s_ of all nanocomposites.

Fig. 8a–d shows the results of CV measurements of all synthesized samples taken at scan rates ranging from 5 to 100 mV s^−1^, which were used to understand the surface redox processes and capacitive behaviour. The semi-circular pattern of the CV profile confirmed that the synthesized electrodes were pseudocapacitive. As the scan rate escalated, the redox peaks exhibited enhanced current responses and expanded integrated areas of the curves. The elevated current response indicates reduced internal resistance of the materials, resulting in greater conductivity, hence corroborating the *I*–*V* study.^21,38^ The measured CV curves indicate that the reductive peak has migrated to greater negative potential values. The oxidative peak changed to higher potential values as the scan rate increased. This phenomenon may be attributed to the heightened internal diffusion resistance. Redox peaks seen even at higher scan rates indicate good capacitive characteristics in the synthesized nanomaterials. The consistency of the peak shapes revealed the favourable reversibility of the redox reaction, implying superior electrode performance. To determine the specific capacitance (*C*_s_) of the manufactured electrode, the following relation was used;^39^5
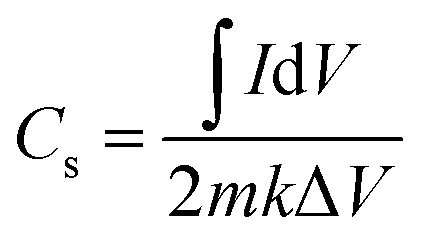
In this case, ‘*k*’ denotes the sweeping rate, ‘*I*d*V*’ stands for the area of the CV loop, ‘*m*’ for the loaded mass, and *V* for the potential window. The incorporation of NrGO and PANI enabled the facile insertion or removal of electrons and ions at the surface of the ternary composite, which exhibited a purely faradaic reaction as indicated by the following chemical equation;

6 ZnS + KOH → ZnSOH + e^1−^ + K^1+^

By providing enough active sites for charge accumulation, the porous structure of the manufactured electrodes significantly improves energy storage performance, hence directly influencing the rise of *C*_s_. Standard electrochemical equation^40,41^ were used to compute the *C*_s_ values of the many electrode materials, ZnS, ZnG, ZnP, and ZnP/NrGO, and found to be 981.11 F g^−1^, 1431.20 F g^−1^, 2151.17 F g^−1^, and 2915.37 F g^−1^, respectively, at a scan rate of 5 mV s^−1^. Reported values for NrGO, PANI have capacitance 234 F g^−1^, 606 F g^−1^.^22,42^ Among these, the ternary composite ZnPG demonstrated the highest specific capacitance, significantly outperforming the binary and pristine counterparts. The major contribution of PANI is faradaic pseudocapacitance associated with the redox reversible transition between leucoemeraldine, emeraldine and pernigraniline formations. The strong redox currents in the CV curves of the electrodes of ZnP and ZnPG (Fig. 8(c and d)) affirm the active role of PANI in the storage of charge. NrGO is mainly used as a high conductive carbonaceous network, with fast electron transfer networks, and inhibition of agglomeration of ZnS nanoparticles. The relevance of the sheet-like form of NrGO, which allows the surface area to be multiplied in a 2-dimensional manner, and allows penetration of the electrolyte, contributing to EDLC. SEM studies show that the synergistic interaction of the conductive, amorphous, and porous character of both PANI and NrGO accounts for his exceptional improvement. The combination of these elements not only enables effective ion diffusion and charge transfer but also expands the electrochemically active surface area, therefore enhancing the general capacitive behaviour of the electrode material.The comparative *C*_s_ values for every electrode arrangement are shown in Fig. 8e. The specific capacitance drops with higher scan rates, as predicted, which is ascribed to the restricted diffusion time for electrolyte ions to reach the inner active sites of the electrode. Higher scan rates show lower charge storage efficiency because of inadequate ion penetration and use of the accessible surface area, which this phenomena causes.^26^

### 5.2 Galvanostatic charging–discharging (GCD)

**Fig. 9:**
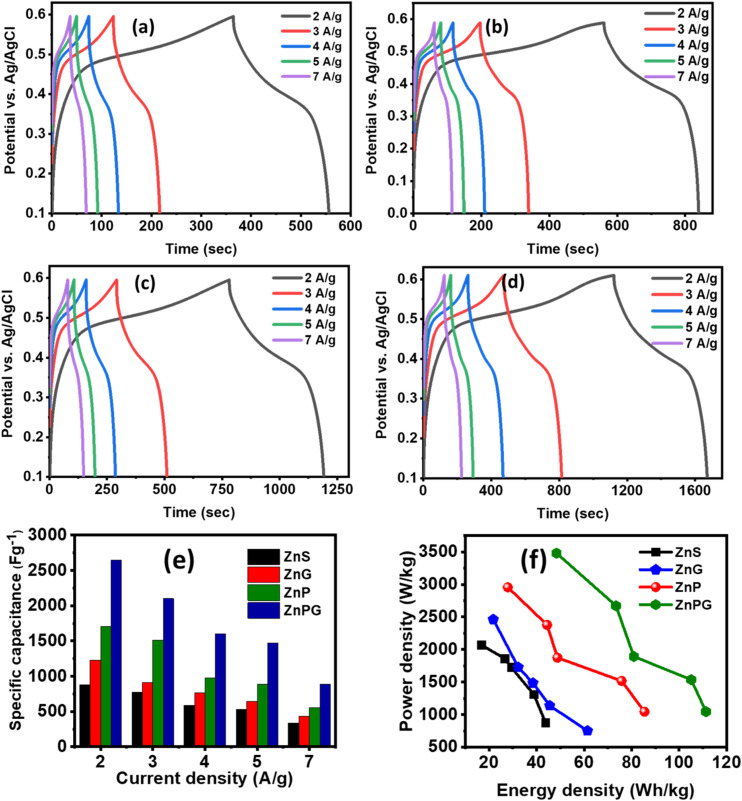
(a–d) GCD curve (e) *C*_s_ at various current densities, (f) Ragone plots.

GCD analysis was conducted within a potential window of 0.1–0.6 V at varying current densities (2–7 A g^−1^) to assess the capacitive behavior of the fabricated electrodes (Fig. 9a–d). The near-symmetric triangular GCD profiles, consistent with the pseudocapacitive characteristics observed in CV analysis, confirm efficient charge storage kinetics.^43^ A minor initial voltage drop during discharge indicates low internal resistance and excellent electrical conductivity of the material.^44^ As shown in Fig. 9e, the *C*_s_ decreased with increasing current density, a trend attributed to limited electrolyte ion diffusion at higher rates. Under rapid charge–discharge conditions, only surface-active sites participate in faradaic reactions, while bulk electroactive regions remain underutilized due to insufficient ion penetration time. This kinetic limitation restricts full access to the electrode's redox-active sites, reducing overall capacitance at elevated current densities.^45^

The *C*_s_, *P*_d_, and *E*_d_ were computed by applying the relation,^46,47^7
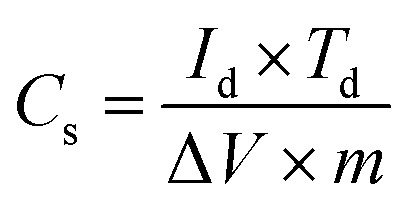
8
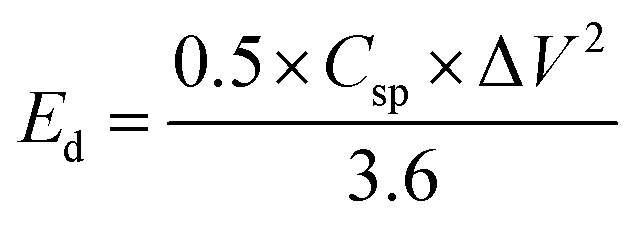
9
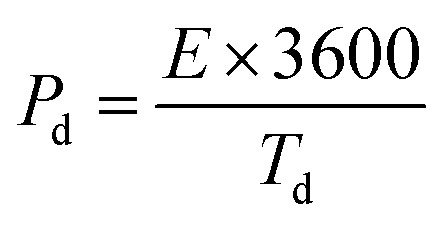


As shown in Fig. 9e, the *C*_s_ decreased with increasing current density, a trend attributed to limited electrolyte ion diffusion at higher rates.

The energy storage characteristics of the synthesized composites were systematically evaluated through Ragone plot analysis (Fig. 9f). The ternary composite demonstrated superior *E*_d_ and *P*_d_ compared to binary counterparts, which can be attributed to three key factors, its hierarchical porous architecture facilitating efficient ion diffusion, the synergistic integration of conductive PANI and NrGO networks with ZnS nanoparticles, and optimized interfacial charge transfer kinetics. The composite maintained excellent rate capability while increasing current density resulted in a gradual decrease in *C*_s_, a characteristic behavior of pseudocapacitive materials due to restricted ion penetration depth, indicating robust electrochemical stability under varied operating conditions.^48^ The comparison of the *C*_s_ of our work and literature is listed in Table 3.

**Table 3:** Comparison of *C*_s_, *E*_d_, *P*_d_ of ZnPG with reported materials for three electrode electrochemical analysis

S. no.	Materials	Methodology	Electrolyte	Specific capacitance (F g^−1^)	Energy density (Wh kg^−1^)	Power density (W kg^−1^)	Ref.
1	ZnS/G	Solvothermal	6 M KOH	197.1	15.6	—	31
2	ZnS//PANI@AC	Hydrothermal	1 M KOH	511.32	32	800	49
3	ZnS/Cu	Solvothermal	3 M KOH	545	—	—	50
4	ZnS-PANI	Co-precipitation and chemical oxidative-polymerization	1 M H_2_SO_4_	893.75	178	300	1
5	NiS/ZnS	Hydrothermal	2 M KOH	1500	50	2165	51
6	Mn/Zns	Hydrothermal	1 M KOH	263	9.12	249	13
7	ZnS	Hydrothermal	1 M KOH.	781	56	200	52
8	ZnPG	Hydrothermal	2 M KOH	2645.94	118	3500	Current work

### 5.3 EIS analysis

**Fig. 10:**
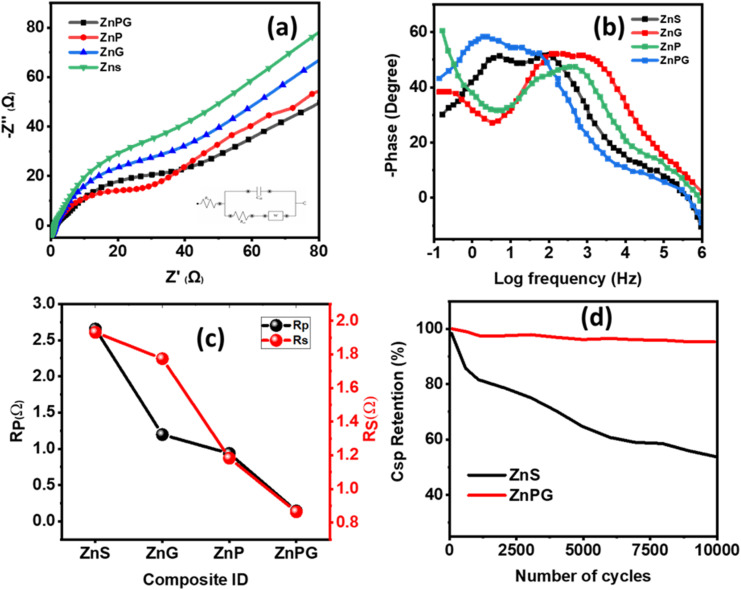
(a) Nyquist plots along with equivalent circuit modeling and (b) Bode's plot. (c) Polarization resistance (*R*_p_) and solution resistance (*R*_s_) values. (d) Stability graph of ZnS and ZnPG composites.

EIS was conducted over the frequency ranging from 0.1 Hz to 1 MHz, and the combined profile of all the fabricated electrodes is depicted in Fig. 10. Nyquist plot illustrates that ZnPG-based electrode exhibits a small semicircular pattern at high-frequency region followed by a nearly linear tail in the low-frequency region which confirms small *R*_p_ and small *R*_s_,^53^ as represented the Nyquist plot in Fig. 10(a). Fig. 10a demonstrates a prominent segment with a high frequency, characterized by a semi-circular form, as well as a part with a low frequency that seems linear. The recorded resistance values for all composites shown in Fig. 10c. The Nyquist plot indicates that the ZnPG nanocomposite exhibits a more restricted semicircle, implying a reduction in interfacial resistance during charge transfer. This feature endows the material with remarkable electrical conductivity. The resistance that emerge from the interfaces of the electrode–electrolyte interface are ascribed to the point of convergence of the low-frequency linear segment, often denoted as “*R*_s_,” and used for quantifying the equivalent series resistance. The polarization resistance (*R*_p_), reflects the interfacial electron transfer kinetics at the electrode–electrolyte interface. Both parameters were extracted by fitting the Nyquist plots using an equivalent circuit model. The relatively low resistance (*R*_s_) value of the ZnPG material contributed to the efficient diffusion of electrolyte ions inside the material. Fig. 10(b) depicts the correlation between the phase angle and logarithmic frequency. Typically, the phase angle of a supercapacitor device tends to approach 90° at low frequencies, indicating a mostly capacitive behavior.^54^ The findings of the study suggest that the curve of the ZnPG material at a 90° has a capacitive behavior in comparison to other materials that were fabricated. The observed phenomenon may be ascribed to the low interfacial resistance and quick ionic kinetics, which are enhanced by ZnPG. The EIS findings indicate that the ZnPG interfacial areas are important in determining the charge-transfer rate and charge-store ability. The conductive NrGO network synergistically combined with pseudocapacitive PANI and electroactive ZnS causes a significant decrease in interfacial resistance, a faster rate of electron/ion movement and an enhanced overall electrochemical activity. These interfacial effects play a major role in the high specific capacitance, rate ability and cycling stability that have been recorded with the ZnPG electrode. The results of the experiment suggest that the conductivity of ZnPG is higher in comparison to the other materials investigated in this research.

### 5.4 Stability test

Cycling stability represents a critical parameter for assessing the practical applicability of electrode materials in industrial settings.^19,27^ To evaluate long-term performance, the ternary composite was subjected to 10 000 galvanostatic charge–discharge cycles at 3 A g^−1^. As shown in Fig. 10d, the composite exhibited outstanding cycling stability with only 4.8% capacitance loss, retaining 95.2% of its initial capacity, while ZnS retained 78.9% capacitive retention. The minimal performance degradation observed during extended cycling demonstrates the material's exceptional structural integrity and electrochemical durability. These results confirm that the ZnPG composite possesses the necessary stability characteristics for real-world energy storage applications.

## 6. Physical characterizations after degradation

**Fig. 11:**
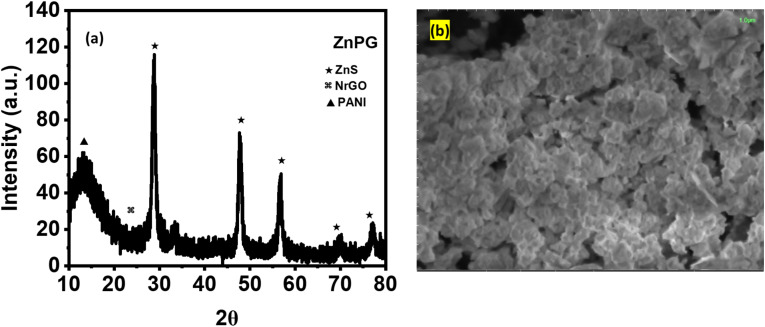
(a) XRD and (b) SEM image of ZnPG after degradation.

The XRD of the ZnPG electrode at the end of 10 000 cycles (Fig. 11a) indicate that neither a significant phase change nor a loss of peaks can be observed, which confirms the structural stability of ZnS at repeated redox cycles. The effect is a modest decrease in the peak intensity and a slight broadening of the peptides which can be explained by partial amorphization or surface rearrangement due to continuous ion insertion/extraction of the sample during cycling. These minor structural adjustments can result in partial loss of electroactive sites that can explain the minor performance decay.SEM image (Fig. 11b) depicted small surface roughening and a localized compaction of particles that is probably related to the repeated ion insertion/extraction and volumetric expansion/contraction of the PANI chains during redox cycling. These small morphological transformations can partially limit access to electrolytes of some active sites, which is one of the reasons that cause the small capacitance decay 4.8% of long-term cycling. Nevertheless, the conserved porous structure maintains prolonged diffusion routes of ions and effective electron transportation. SEM and XRD analysis shows that the ZnPG composite is highly morphologically robust, and the low level of structural evolution confirms the synergistic ZnPG interfacial network is effective in sustaining an electrochemical performance after several cycles of mechanical activity.

## 7. Asymmetric electrochemical analysis

**Fig. 12:**
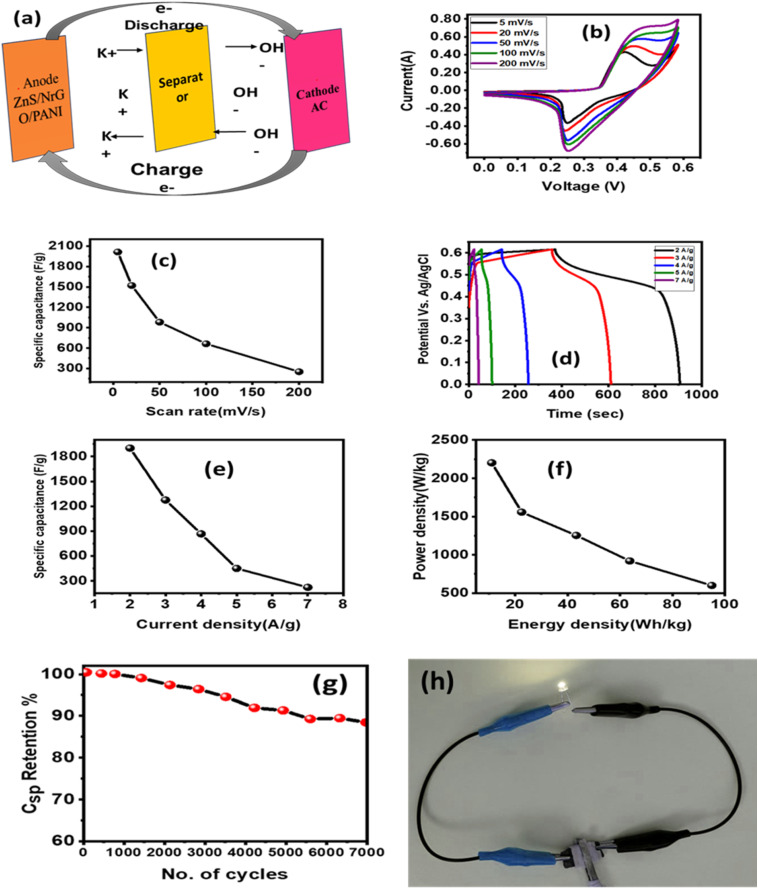
(a) Asymmetric device schematic, (b) CV curves, (c) scan-rate-dependent *C*_s_, (d) GCD profiles, (e) current-density-dependent *C*_s_, (f) Ragone plot, (g) cycling stability, and (h) LED light on of ZnPG electrode.

Fig. 12a presents the schematic design of the asymmetric supercapacitor configuration. The redox characteristics were evaluated through CV measurements (Fig. 12b). As shown in Fig. 12c, the ternary composite exhibited scan-rate-dependent capacitance behavior, with *C*_s_ values decreasing from 2013.15 F g^−1^ at 5 mV s^−1^ to 251.82 F g^−1^ at 200 mV s^−1^. GCD analysis in the two-electrode configuration (Fig. 12d) revealed outstanding electrochemical performance, with the composite achieving 1901.51 Fg^−1^ at 2 Ag^−1^. The observed capacitance reduction at higher current densities (Fig. 12e) results from insufficient electrolyte penetration time at elevated charge/discharge rates.

For practical applications, the *E*_d_, *P*_d_ and cycling stability represent crucial performance metrics. The Ragone plot in Fig. 12f demonstrates the device's energy-power characteristics, while the cycling test (Fig. 12g) shows excellent capacitance retention of 88.71% after 7000 cycles. Although the two-electrode configuration shows lower *C*_s_, *E*_d_, and *P*_d_ values compared to three-electrode measurements, the practical demonstration in Fig. 12(h) confirms the device's viability – two series-connected supercapacitors successfully illuminated an LED for over 58 seconds.

## 8. Conclusion

ZnS and its composites (ZnG, ZnP, ZnPG) were synthesized *via* the hydrothermal method. ZnPG nanocomposites were engineered to exhibit exceptional pseudocapacitive behavior, achieving a remarkable *C*_s_ of 2645.94 F g^−1^ at 5 mV s^−1^ and outstanding cycling stability (95.2% retention after 10 000 cycles). The ternary composite's hierarchical porous structure, enhanced electrical conductivity, and synergistic interfacial interactions between ZnS, NrGO, and PANI contributed to its superior energy storage capabilities. In an asymmetric supercapacitor configuration, the ZnPG electrode delivered a high capacitance of 1901.51 F g^−1^ at 2 A g^−1^, along with impressive *E*_d_ (95.05 Wh kg^−1^) and *P*_d_ (2201.21 W kg^−1^). A practical demonstration confirmed its viability, powering an LED for 58 seconds. These results highlight the composite's potential as a high-performance, cost-effective alternative to conventional electrode materials. The findings underscore the significance of ternary nanocomposites in advancing next-generation energy storage technologies, addressing the growing demand for efficient, durable, and sustainable supercapacitor materials. Future research should focus on optimizing synthesis parameters and scaling up production for commercial applications.

## Author contributions

M. U. Islam and Javed Ahmad coined idea of the work and supervised. Maria Sadiq synthesized the materials, analyzed the data and wrote original draft under. Maryam Hina, M.Salman, Mamoona Nawaz provides consultancy to carry out the work and assisted to proofread the manuscript. All authors have approved the final version of the manuscript.

## Conflicts of interest

The authors declare that they have no known competing financial interests or personal relationships that could have appeared to influence the work reported in this paper.

## Data Availability

All the related data is available from the authors upon request.
